# Influence of Electrode Placement on Signal Quality for Ambulatory Pregnancy Monitoring

**DOI:** 10.1155/2014/960980

**Published:** 2014-02-03

**Authors:** M. J. Rooijakkers, S. Song, C. Rabotti, S. G. Oei, J. W. M. Bergmans, E. Cantatore, M. Mischi

**Affiliations:** ^1^Faculty of Electrical Engineering, University of Technology Eindhoven, 5612 AZ Eindhoven, The Netherlands; ^2^Máxima Medical Center, 5504 DB Veldhoven, The Netherlands

## Abstract

Noninvasive fetal health monitoring during pregnancy has become increasingly important in order to prevent complications, such as fetal hypoxia and preterm labor. With recent advances in signal processing technology using abdominal electrocardiogram (ECG) recordings, ambulatory fetal monitoring throughout pregnancy is now an important step closer to becoming feasible. The large number of electrodes required in current noise-robust solutions, however, leads to high power consumption and reduced patient comfort. In this paper, requirements for reliable fetal monitoring using a minimal number of electrodes are determined based on simulations and measurement results. To this end, a dipole-based model is proposed to simulate different electrode positions based on standard recordings. Results show a significant influence of bipolar lead orientation on maternal and fetal ECG measurement quality, as well as a significant influence of interelectrode distance for all signals of interest.

## 1. Introduction

Monitoring of the fetal heart rate (fHR), fetal electrocardiogram (fECG) morphology, and uterine activity is important for fetal health assessment during pregnancy and delivery. Current monitoring methods are, however, not suited for long-term observation throughout pregnancy. The most commonly used method for fetal monitoring during pregnancy uses Doppler ultrasound and a strain gauge to determine the fHR and uterine activity, respectively. While allowing for noninvasive measurements, Doppler ultrasound measurements need continuous attention of a trained physician, making them unsuitable for long-term observation [1]. Furthermore, in many situations these measurements do not provide conclusive information for accurate assessment of fetal health and, therefore, additional information is needed for clinical decision making [2]. During delivery, a fetal scalp electrode and an intrauterine pressure catheter (IUPC) can be used as part of an internal cardiotocogram (CTG) [3]. Although this method provides accurate fECG and IUP readings, thus improving decision making, it requires rupturing of the membranes; it can, therefore, be applied during delivery only.

Recently published methods enable noninvasive extraction of the fECG from abdominal recordings, the results of which have shown to be more reliable and accurate than ultrasound [4–11]. Additionally, based on the electrohysterogram (EHG), which is a recording of the electrical activity causing uterine muscle contractions, an intrauterine pressure (IUP) estimate can also be obtained [12, 13]. As both methods use abdominal contact electrodes, a single set of electrodes can enable prolonged noninvasive monitoring of both fECG and IUP. This allows for determination of the fetal health and onset of labor throughout pregnancy with a relatively high patient comfort.

Numerous electrode configurations have been presented for both fECG and EHG measurements, using a wide variety of leads ranging in numbers from only a few to one hundred [6, 7, 12, 14–17]. Although abdominal electrode placement determines the measured signal quality to a great extent, no consensus has been reached on standard electrode placement. Because of the prolonged measurement periods in ambulatory monitoring, comfort in placing and wearing the sensors is important, imposing additional requirements on the electrode spacing and number of sensors used. Also, the number of sensors greatly influences the total power consumption of the monitoring system, which is a major concern in ambulatory monitoring [15, 18].

The goal of this paper is to determine an electrode grid that allows for a dynamic selection of the optimal electrode positions for an assessment of the fetal health and onset of labor in an ambulatory setting. More specifically, we focus on determination of the fetal heart-rate and uterine contraction frequency near the end of the pregnancy, when monitoring these parameters is more relevant. To this end, the influence of abdominal electrode placement on the signal quality of the fetal and maternal electrocardiogram (ECG) as well as the EHG is explored. Various bipolar electrode placements are evaluated based on both simulated and measured signals to determine the influence of scale and direction on measurement quality for all signals of interest. For simulation, a dipole-based model is used to calculate the potential on the thoracic surface, with a maternal and fetal vector cardiogram (VCG) as signal sources. These VCGs can be extracted from any standard thoracic or abdominal ECG recording, respectively, to simulate a multitude of electrode placements. For the measurements, an electrode placement protocol is employed that enables measurement of the maternal and fetal ECG as well as the EHG from a plurality of bipolar leads in multiple directions and interelectrode scales. A bipolar setup is used because it reduces the common mode interference, while enhancing the relative contribution of the signals of interest, depending on the lead orientation [6, 19]. Both simulations and measurements are used to determine the influence of electrode placement on the recorded signal quality, defined by its signal-to-noise ratio (SNR). Results show that both electrode orientation and distance significantly influence the measurement quality of the signals of interest.

First, an introduction of all physiological parameters influencing abdominal measurements is presented in Section 2. Next, Section 3 outlines a strategy to find an optimal electrode grid for ambulatory fetal health monitoring, where a model for simulation of the maternal and fetal ECG is proposed in Section 3.1, followed by the measurement setup description in Section 3.2. Finally, Section 3.3 introduces all criteria for choosing electrode positions for an optimal electrode grid. The simulation and measurement results are shown in Section 4.  Section 5 contains a discussion on the obtained results and provides guidelines for choosing an optimal electrode grid.

## 2. Physiological Parameters

Various physiological signals play an important role in pregnancy monitoring. The fECG can be used to determine the fHR with beat-to-beat accuracy, but it also allows for analysis of the fECG morphology [7]. The EHG on the other hand can be used to estimate the IUP and recognize the first signs of approaching delivery [20]. During labor, the fHR can then be used in combination with the EHG. The pressure exerted on the fetus by the contracting uterus acts as a stress test, and the fetal heart rate variability gives an indication of the level of fetal distress [21]. Multiple noise sources are also present in abdominally measured signals, the most noticable of which are the maternal electrocardiogram (mECG) and electromyographic signals from the abdominal muscles [22]. A list of the main signals that are present in abdominal recordings is presented hereafter.

### 2.1. Fetal ECG

The fECG is a representation of the potential map in the fetal heart resulting from de- and repolarization of myocytes over time. For a fetus with a gestational age between 32 to 41 weeks, the fetal HR is in the range of 60–240 BPM with an expected HR around 140 BPM, while a higher instantaneous HR is possible due to arrhythmias [23]. Most of the QRS spectral energy is contained in the 20–60 Hz range, with a peak-to-peak amplitude in the range of 3–25 *μ*V.

### 2.2. Electrohysterogram

The EHG is a bioelectric signal associated with the propagation of action potentials through uterine muscle cells. It is the primary cause of uterine muscle contractions. Therefore, the EHG can be used as an estimator of uterine activity and permits estimation of the IUP associated with each contraction [12]. Contrary to the myocardium, the action potential waves propagating through the uterus do not have a fixed pacemaker location, resulting in an erratic direction of propagation. Research has shown that the main EHG frequency components are in the 0.3–0.8 Hz band with a peak-to-peak amplitude >100 *μ*V [14, 20, 24].

### 2.3. Maternal ECG

The mECG is generally the most prominent signal present in abdominal ECG recordings. Therefore, the mECG needs to be removed to enable reliable fECG detection. This procedure typically requires accurate detection of the maternal R-peaks and delineation of the the P-wave, QRS complex, and T-wave [7]. For pregnant women, the HR can range from 50 to 210 BPM with an expected HR around 80 BPM, while most of the QRS spectral energy is contained in the 10–30 Hz frequency range [25, 26]. Signal amplitude and morphology both depend on electrode placement. The amplitude is in the order of 100 *μ*V when measured on the abdomen, which is 4–20 times that of the fECG [6, 27].

### 2.4. Additional Noise Sources

Abdominal ECG and EHG recordings are affected by various noise sources. Apart from the mECG, abdominal recordings can contain various high-amplitude noise sources, which are typically highly nonstationary in time and colored in spectrum [28, 29].

*Skeletal muscle artifacts* introduce high frequency components between 10 Hz and 500 Hz during skeletal muscle activity, when recorded abdominally. Inparticular during a contraction, the skeletal muscle electromyogram can be regarded as the major noise contribution [24, 30].

*Electrode motion artifacts* are transient baseline changes which originate from changes in the electrode-skin impedance and changes of skin potential due to deformation of the skin [31]. On average, electrode motion artifacts are 100–500 ms in length with an amplitude of up to five times that of the mECG [29].

*Fetal movements* can cause skeletal muscle artifacts, and more importantly, can result in a different orientation of the fetal heart vector with respect to the electrode grid, changing the amplitude and morphology of the measured signals [32].

*The vernix caseosa* is a thin fatty layer which starts developing on the fetal skin from the 28th week of gestation, changing the conductivity in the maternal volume conductor. This severely reduces the measured fECG amplitude until the vernix caseosa partly dissolves, which typically starts in the 32nd week of gestation [33].

## 3. Methodology

In an effort to determine the optimal electrode placement for ambulatory fetal health monitoring, the influence of electrode placement on the signal quality of the mECG, fECG, and EHG is determined. To this end, an electrophysiological model for simulation of the abdominal skin potential and a set of abdominal measurements are introduced.

The model described in Section 3.1 can simulate the electrical potential on the body surface as a result of electrical field vectors of the maternal and fetal hearts. This model can be used to simulate any electrode configuration on the maternal thorax based on standard thoracic and abdominal measurements and used to determine and verify the optimal measurement positions for both mECG and fECG. A similar simulation of the EHG is not possible due to the unknown origin and propagation direction of the signal [34].

Section 3.2 describes a set of abdominal measurements and a method of extracting bipolar leads at various interelectrode scales and in various orientations. These bipolar leads allow for comparison of the relative signal quality of all ECG and EHG signals at different positions and orientations.

Section 3.3 defines the criteria for selection of optimal electrode sets for maternal and fetal ECG as well as EHG measurement based on their SNR. The leads with the highest SNR for each patient are selected based on the results for both simulated and measured signals. Here, the SNR calculation of the EHG signal is based solely on abdominal measurements. The statistical significance of the SNR improvement over the other bipolar leads is determined based on the *P* value to determine which changes in direction and interelectrode distance result in significant SNR reduction.

### 3.1. Electrophysiological Model

#### 3.1.1. The Maternal Heart

**Figure 1:**
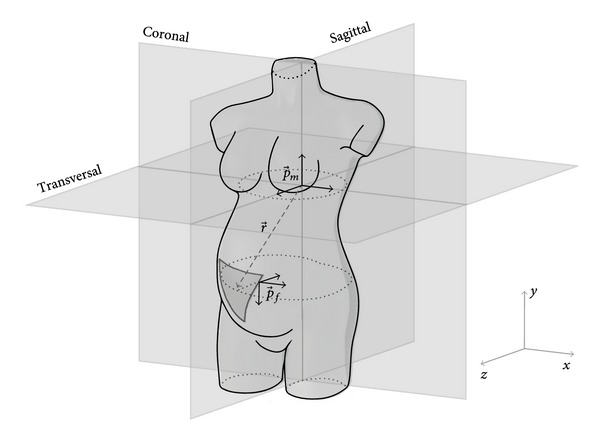
Relative locations of the maternal and fetal VCGs ${\vec{p}}_{m}$ and ${\vec{p}}_{f}$, the placement of electrodes on the maternal abdomen indicated by the triangular region, and the vector connecting both $\vec{r}$.

Cardiac contractions are the result of the generation and conduction of electrical pulses, referred to as action potentials, through the myocardium. Propagation of these action potentials acts as the movement of numerous electrical dipoles, which can be modeled as a single dipole. This results in an electrical cardiac field vector $\vec{p}$ [35, 36], which can be represented as

(1) $$\begin{array}{c} \vec{p}=\overset{N}{\underset{i=1}{\sum }}q_{i}{\vec{d}}_{i}=p_{x}{\vec{a}}_{x}+p_{y}{\vec{a}}_{y}+p_{z}{\vec{a}}_{z}, \end{array}$$

where *q*_*i*_ and *d*_*i*_ are the size of the *i*th charge and its distance from the origin, respectively, and ${\vec{a}}_{x}$, ${\vec{a}}_{y}$, and ${\vec{a}}_{z}$ are three orthonormal vectors related to the three body axes. This cardiac vector originates in the center of the heart and varies over time in both amplitude and orientation defining a specific trajectory, which is referred to as the VCG. Using the Moore-Penrose inverse of the Dower matrix [37, 38], also called the inverse Dower transform, it is possible to extract $\vec{p}$ from a standard 12-lead ECG [39, 40]. The inverse Dower transform takes into account the standard locations of the recording leads and the attenuation of the body volume conductor for each electrode; it is defined as

(2) $$\begin{array}{c} \left[\begin{array}{c} p_{x}\\ p_{y}\\ p_{z} \end{array}\right]={\left[\begin{array}{ccc} -0.172 & 0.057 & -0.229\\ -0.074 & -0.019 & -0.310\\ 0.122 & -0.106 & -0.246\\ 0.231 & -0.022 & -0.063\\ 0.239 & 0.041 & 0.055\\ 0.194 & 0.043 & 0.108\\ 0.156 & -0.227 & 0.022\\ -0.010 & 0.887 & 0.102 \end{array}\right]}^{T}\left[\begin{array}{c} V_{1}\\ V_{2}\\ V_{3}\\ V_{4}\\ V_{5}\\ V_{6}\\ D_{I}\\ D_{II} \end{array}\right], \end{array}$$

where *V*_1_–*V*_6_ are the six precordial leads and *D*_*I*_ and *D*_*II*_ are Einthoven leads *I* and *II*, respectively. The resulting cardiac vector $\vec{p}$ can now be obtained from a standard ECG recording and used to simulate the potential map on the skin surface. Assuming the body volume conductor to be a homogeneous conductor, a linear projection of $\vec{p}$ can be used to estimate the ECG signal. In case of a homogeneous infinite-volume conductor, the potential *v* on the body surface generated by $\vec{p}$ is defined by the simplified model

(3) $$\begin{array}{c} v=\frac{1}{4\pi \sigma }\frac{\vec{p}\cdot \vec{r}}{{\left|\vec{r}\right|}^{3}}, \end{array}$$

where $\vec{r}=r_{x}{\vec{a}}_{x}+r_{y}{\vec{a}}_{y}+r_{z}{\vec{a}}_{z}$ is the vector connecting the origin of the cardiac vector $\vec{p}$ with the electrode location and *σ* is the conductivity constant of the conductive medium.  Figure 1 shows the relative locations of both the maternal and fetal cardiac vector, ${\vec{p}}_{m}$ and ${\vec{p}}_{f}$, the approximate electrode placement on the maternal abdomen, and $\vec{r}$ connecting both.

**Figure 2:**
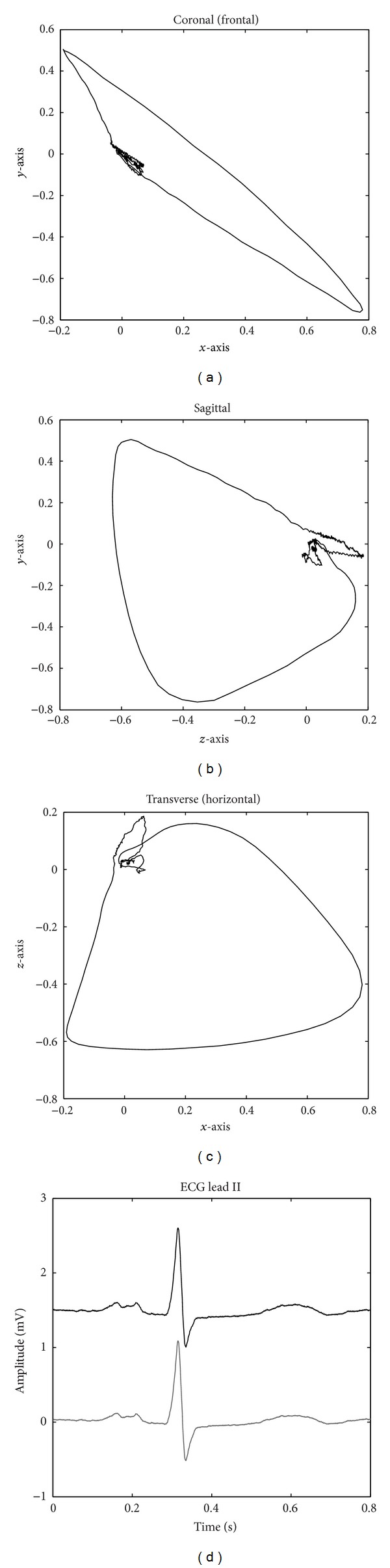
(a)–(c) show the *x*-, *y*-, and *z*-axes of a normal VCG, respectively. (d) shows the original measured thoracic Lead II (top) and the simulated version (bottom) using the dipole model and the estimated VCG.

The model can now be used to calculate the potential of a simulated ECG anywhere on the thorax using the VCG obtained from a standard 12-lead ECG. Here, recordings I12 and I32 from the St. Petersburg INCART 12-lead Arrhythmia Database were selected for maternal VCG extraction based on gender and age of the subjects. Nonarrhythmic episodes with a total length of 30 min were selected for the female patients, 39 and 41 years of age.  Figure 2 shows the coronal, sagittal, and transverse projections of a VCG extracted from recording I12, as well as a plot of Einthoven lead II, both measured and simulated using (3).

#### 3.1.2. The Fetal Heart

**Figure 3:**
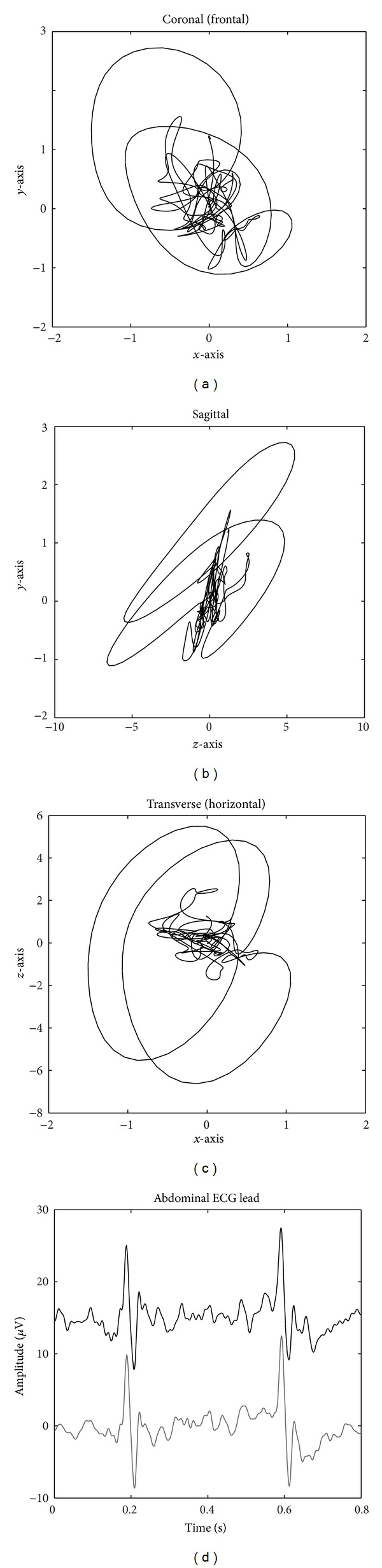
(a)–(c) show the *x*-, *y*-, and *z*-axes of a normal fVCG, respectively. (d) shows the original measured lead (top) 2nd lead in [7] and the simulated version (bottom) using the dipole model and the estimated fVCG.

The fetal heart model is similar to that of the maternal heart with the exception of its position and orientation relative to the electrodes. However, the used fetal VCG (fVCG) differs from the VCG used for simulation of the maternal cardiac activity. A matrix operation similar to the inverse Dower matrix in (2) can be used to extract the fVCG from the abdominal electrodes. To this end, the matrix coefficients are adjusted based on the spatial locations of the electrodes with respect to the fetal heart, as discussed in [32]. For the measurement setup as described in [7], the adjusted matrix to obtain the fVCG ${\vec{p}}^{f}$ is defined as

(4) $$\begin{array}{c} \left[\begin{array}{c} p_{x}^{f}\\ p_{y}^{f}\\ p_{z}^{f} \end{array}\right]={\left[\begin{array}{ccc} -1.074 & -1.200 & 1.044\\ -0.715 & -0.615 & 1.287\\ -0.650 & 0.604 & 1.280\\ -1.095 & 1.196 & 1.040\\ -1.309 & 1.200 & -0.528\\ -0.507 & 0.615 & -1.038\\ -0.578 & -0.606 & -1.003\\ -1.266 & -1.198 & -0.529 \end{array}\right]}^{T}\left[\begin{array}{c} L_{1}\\ L_{2}\\ L_{3}\\ L_{4}\\ L_{5}\\ L_{6}\\ L_{7}\\ L_{8} \end{array}\right], \end{array}$$

where *L*_1_–*L*_8_ are leads 1–8 as described in [7]. Two abdominal measurements with a high SNR measured at a gestational age of 22 weeks and 24 weeks, respectively, were selected from the dataset described in [7] for extraction of a fVCG.  Figure 3 shows the three orthogonal projections of a fVCG obtained from these recordings, as well as a plot of the 2nd electrode (*L*_2_) of the abdominal recording, both measured and simulated. It can be noted that the fVCG is rotated with respect to the visualization planes compared to the maternal VCG, due to the orientation of the fetal body. Additionally, the fVCG is much more erratic due to the lower SNR.

#### 3.1.3. Noise Sources

Reduction of the recorded ECG signals to three dimensions using the inverse Dower transform results in reduction of the noise, as can be observed in Figure 3(d), where the simulated signal is smoother than the original measurement. In these simulations the remaining noise from the original ECG, in fact, originates from the same spatial location as the ECG signal. Therefore, the noise amplitude in the simulation scales at the same rate as the ECG with changing electrode distance. Hence, the SNR will remain equal irrespective of the interelectrode distance. Electrode motion artifacts and skeletal muscle artifacts in real measurements are localized electrical signals and, therefore, the amplitude of these artifacts is not influenced by interelectrode distance. As a consequence, the ECG signal amplitude typically scales up faster with increasing interelectrode distance than the noise, resulting in an increased SNR. As the frequency spectrum of skeletal muscle artifacts shows the largest overlap with that of the fetal and maternal ECG, modeled skeletal muscle noise is added to each of the signals at the simulated electrode positions.

The skeletal muscle artifacts are modeled by coloring white noise based on the typical electromyogram power spectrum. The filter used for noise coloring is a 2nd order band-pass filter combined with a low-pass filter tuned to match the recorded power spectrum in [30]. As the amplitude of artifacts due to fetal movements scales with changing interelectrode distance, this type of noise is not added to the model.

The modeled skin potential *v*_*o*_ can now be described for each electrode by

(5) $$\begin{array}{c} v_{o}\left(t\right)=v_{m}\left(t\right)+v_{f}\left(t\right)+\alpha \cdot \left(h*w\right)\left(t\right), \end{array}$$

where *v*_*m*_ and *v*_*f*_ are the maternal and fetal simulation results, respectively, *h*(*t*) is the impulse response of the skeletal-muscle-artifacts-coloring filter, *w*(*t*) is white noise, *α* is a scaling factor for the skeletal muscle artifacts, and ∗ is the convolution operator. The scaling factor *α* is chosen such that the SNR of the best bipolar lead is comparable to that of recordings as described in Section 3.2.

### 3.2. Abdominal Measurements

A set of test measurements was performed to determine optimal electrode placement for ambulatory fetal monitoring. The dataset contains five abdominal measurements of 20 min each on pregnant women at full gestation (39 weeks and 4 days ± 12 days) with a total of 7,148 maternal and 13,612 fetal heart beats, as well as 35 uterine contractions. Ultrasonography by medical personnel showed all fetuses have a cephalic presentation and no fetuses making any mayor thoracic movements. Simultaneously with each measurement, an external cardiotocogram was recorded to monitor both fHR and uterine activity. All measurements were performed with an ethical approval at the Máxima Medical Center in Veldhoven (The Netherlands), after patients signed an informed consent.

**Figure 4:**
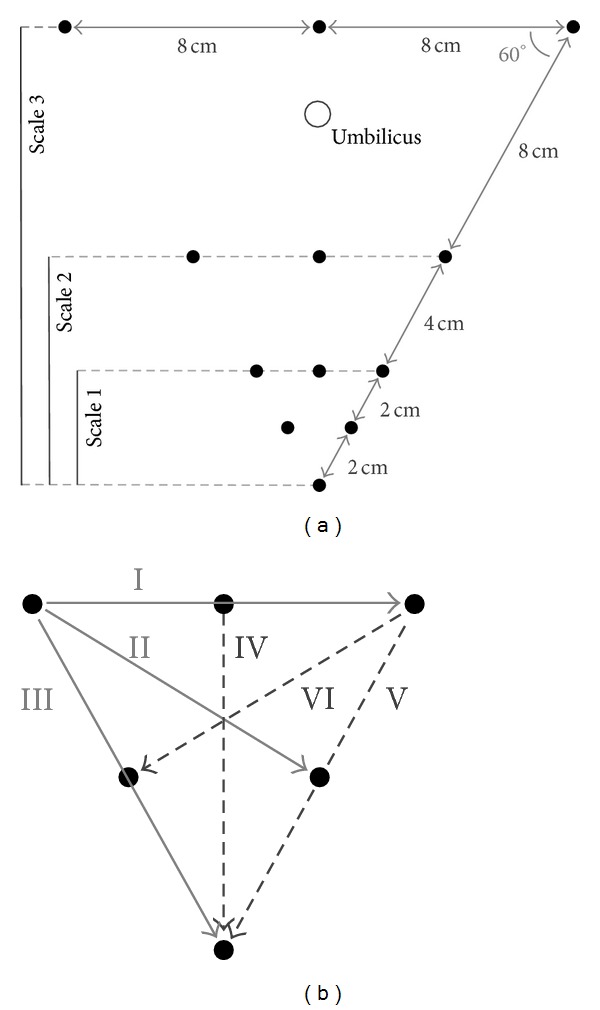
Electrode grid used for test measurements. In (a) the whole electrode grid including the three used measurement scales is shown. In (b) the six different bipolar lead directions for each of the scales are indicated.

All measurements were performed using a Refa system (TMS International, Enschede, the Netherlands), comprising a multichannel amplifier for electrophysiological signals, with a noise level <1 *μ*V RMS and a discretization level of 18.4 nV/bit. An electrode grid with a triangular layout, as shown in Figure 4(a), was used for all measurements. This layout was chosen because a minimum of three electrodes are needed to register a two-dimensional signal, and because it is easily scalable to a different grid size while reusing a large part of the electrodes. In addition to the electrodes shown in Figure 4, ground and reference electrodes were placed on the hip [6, 12]. The triangular grid shown in Figure 4(a) can be used to analyze recorded signals at three different interelectrode scales, each consisting of six electrodes as shown in Figure 4(b). At each of the three indicated scales, bipolar derivations were calculated in 6 different directions, resulting in a total of 18 bipolar signals for each measurement. Because a triangular grid is used to determine bipolar signals at 30° increments, the interelectrode distance within a single scale varies. Bipolar leads II, IV, and VI are a fraction $\delta_{\Delta }=1-\sqrt{3/4}$ shorter than those of leads I, III, and V.

### 3.3. Quality Measures

To define the ability of the electrode grid to capture all electrophysiological characteristics of interest, a signal quality criterion for both the fetal and maternal ECG as well as the EHG is defined.

#### 3.3.1. Maternal and Fetal ECG Quality Measure

An estimate of the maternal and fetal SNR is used as the quality measure for the maternal and fetal ECG, respectively. For the mECG, the abdominal ECG is first preprocessed to only include noise in the same frequency band as the ECG, which can influence ECG detection and extraction results. Band-pass filtering with cut-off frequencies of 2 Hz and 70 Hz is used to remove baseline wander and high-frequency noise. Powerline interference is removed by means of a notch filter. Next, the SNR is calculated for a single maternal QRS complex based on the method described in [41]. This SNR is defined as

(6) $$\begin{array}{c} {\text{SNR}}_{m}=10\cdot \log_{10}\frac{V_{\text{mQRS},\text{PP}}^{2}/8}{\left(1/N\right)\cdot \sum S_{\text{noise}}^{2}}, \end{array}$$

where *V*_mQRS,PP_ is the peak-to-peak voltage of the maternal QRS-complex in a 100 ms wide window around the annotated peak location, *S*_noise_ is the noise signal in the 2 segments between the surrounding QRS-complexes, and *N* is the number of samples in *S*_noise_. Here, the signal power can be derived from the square of *V*_mQRS,PP_ by dividing it by 8, when approximating the QRS complex as a sinusoidal signal [41]. Because *V*_mQRS,PP_ is determined from the measured signal, which includes noise, the calculated SNR defined above is actually a ratio of the signal-plus-noise to the noise and as a consequence is typically over-estimated.

SNR calculations for the fECG are similar to those for the mECG, with the exception of the removal of the mECG prior to fetal SNR (SNR_*f*_) calculation and changes in the band-pass cut-off frequencies. Various methods for removal of the mECG have been presented in the literature [4–9]. As the method proposed by Vullings et al. outperforms the other techniques in both mECG removal and fHR detection, the maternal ECG was removed using dynamic segmentation and linear prediction [7]. Based on the assumption that all maternal ECG components are removed prior to fetal R-peak detection using [32], the amplitude of the maternal R-peaks becomes irrelevant when calculating the fetal SNR. Additionally, the cut-off frequencies of the filter are set at 5 Hz and 100 Hz, respectively, to account for the different spectral content of the fECG. For the calculation of both maternal SNR and fetal SNR, the R-peak locations must be known. To this end, all R-peaks in the dataset have been manually annotated.

#### 3.3.2. EHG Quality Measure

Similar to the ECG, the EHG quality measure is defined by its SNR, where signal and noise segments were defined as contraction and noncontraction segments in the abdominal EHG signal, respectively. The contraction timing was defined as periods with an IUP increase of over 10% compared to the baseline pressure, as measured with the IUPC reference signal. Signal segments with a 10% rise over the baseline shorter than 20 s were rejected. A SNR was obtained for each contraction, as defined by

(7) $$\begin{array}{c} {\text{SNR}}_{\text{EHG}}=10\cdot \log_{10}\frac{\left(1/M\right)\cdot \sum S_{\text{EHG}}^{2}}{\left(1/N\right)\cdot \sum S_{\text{noise}}^{2}}, \end{array}$$

where *S*_EHG_ and *S*_noise_ are the signal and noise power in the 0.3–0.8 Hz frequency band, respectively, in line with [12], and *M* is the number of samples in *S*_EHG_.

## 4. Results

**Figure 5:**
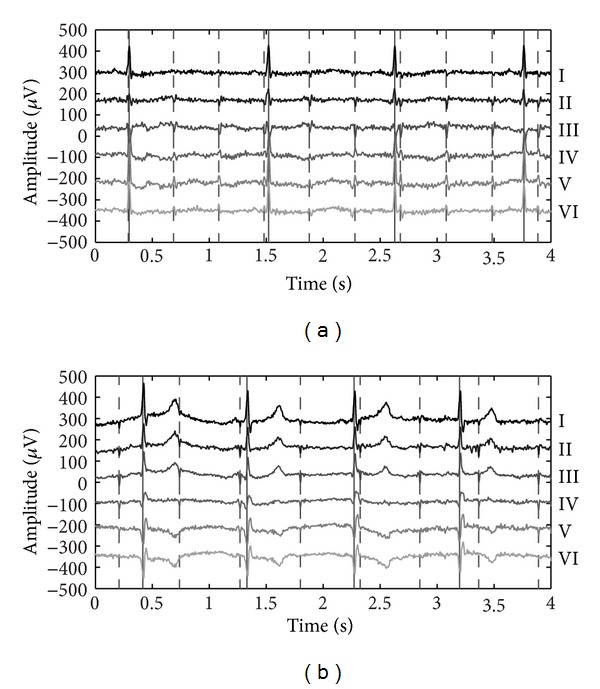
Example of both (a) a simulated and (b) a measured abdominal ECG recording for 6 bipolar leads at the 16 cm scale. Maternal and fetal R-peaks are indicated by solid and dotted vertical lines, respectively.

Figure 5 shows a 4 s segment of both simulation results of the model described in Section 3.1 and a recording as described in Section 3.2, respectively. Both use the electrode grid as introduced in Figure 4(b) with an interelectrode distance of 16 cm. In both plots the mECG is an order of magnitude larger than the fECG and that the orientation of the bipolar measurement influences the amplitude and morphology of both maternal and ECG signals. In both cases, leads I and III are the leads with the largest QRS energy for the maternal and fetal ECG, respectively.

Tables 1 and 2 show the $\bar{\text{SNR}}$ in simulated signals for the maternal and fetal ECG, respectively. Next to the three scales at which measurements were performed, two additional scales with 12 cm and 20 cm interelectrode distance were simulated. The bold faced SNR in each row indicates the bipolar channel with the highest SNR at the current scale, for each of the simulations. The SNR for both mECG and fECG shows an increase with increasing interelectrode distance.

Tables 3 and 4 show the $\bar{\text{SNR}}$, for the maternal and fetal ECG, respectively, for the abdominal measurements as described in Section 3.2. The bold faced SNR in each row indicates the bipolar channel with the highest SNR at the current scale, for each of the patients. Similar to the simulations, the SNR for both maternal and fetal ECG shows a clear increase with increasing interelectrode distance. For the maternal ECG, a clear preferential measurement direction can also be observed.

**Figure 6:**
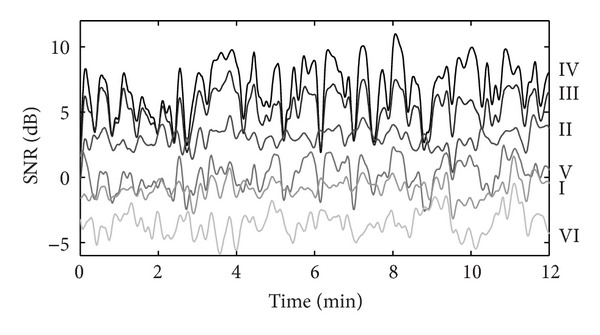
SNR of the fECG in six bipolar derivations in the recording of patient 3.

Figure 6 shows part of the fECG SNR in six bipolar derivations at the 16 cm scale. For display purposes, the SNR has been smoothened taking the running average of the SNR in a 20 s window. Even though the SNR in all leads fluctuates over time, Lead IV clearly has the highest SNR throughout the recording. Leads I and VI, which are at a large angle with lead IV, have the lowest SNR.

**Figure 7:**
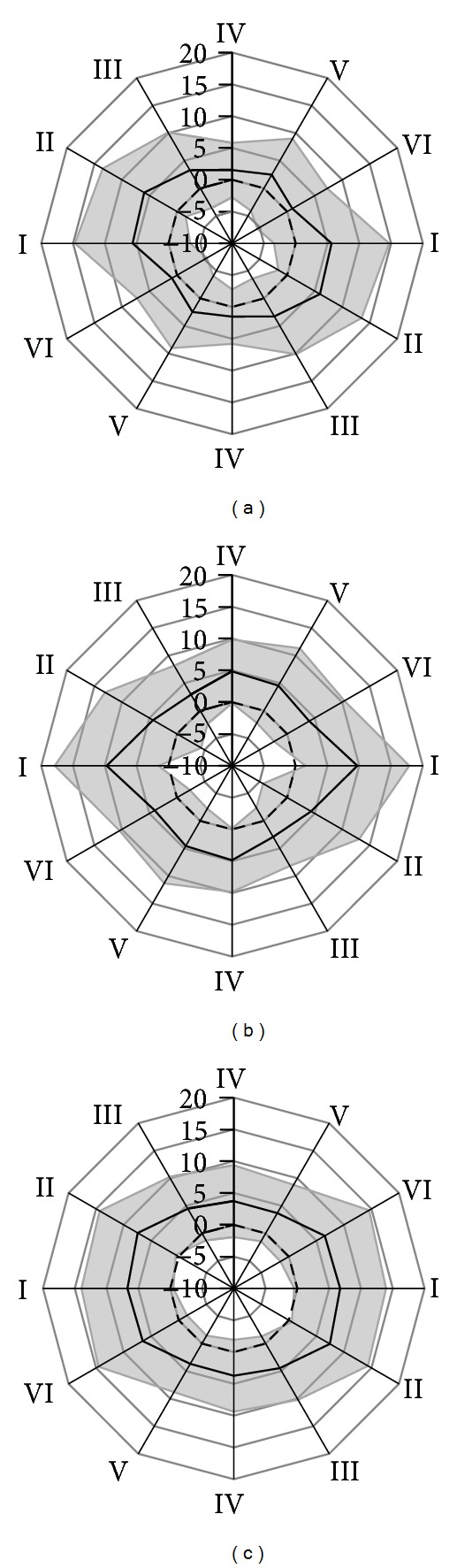
Mean (black line) and standard deviation (grey area) of the SNR of the measured EHG in dB. Bipolar derivations in six directions are shown for interelectrode distances of (a) 4 cm, (b) 8 cm, and (c) 16 cm.

Figure 7 shows the mean and standard deviation of the EHG SNR for each of the six possible bipolar lead orientations and each of the three interelectrode scales. From this figure it can be observed that, on average, a horizontal measurement direction gives the best EHG measurement results, and the SNR with respect to the interelectrode distance contains an optimum between 8 cm and 16 cm.

**Figure 8:**
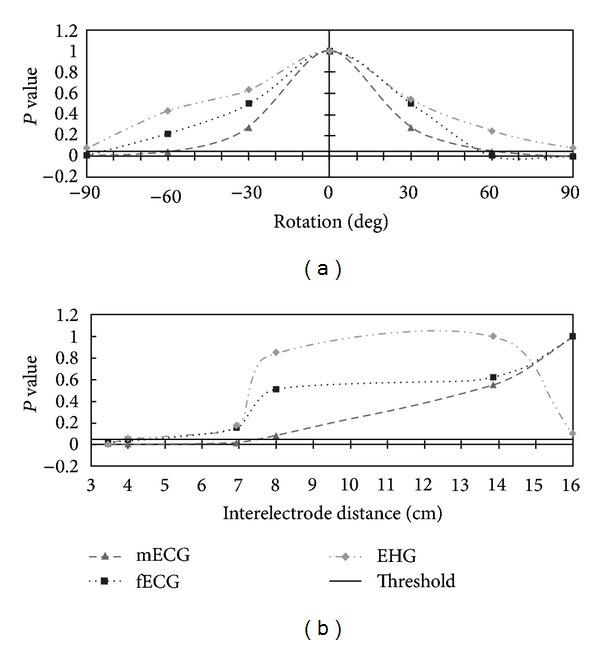
*P* values of the measured mECG, fECG, and EHG signals over a range of settings relative to the optimal value. (a) *P* value as a function of the rotation of the bipolar electrode pair, relative to the optimal electrode orientation over all distances. (b) *P* values as a function of interelectrode distance over all orientations, relative to the optimal distance.

The statistical significance of the obtained signal quality at various measured electrode locations is expressed by its *P* value, obtained from a number of *t*-tests. A value below 0.05 is considered to be statistically significant.  Figure 8 shows the statistical significance of a relative change of interelectrode distance and rotation, with respect to the optimal electrode placement. Additionally, the 0.05-threshold line is shown in both figures indicating which changes in interelectrode distance or orientation are significant.

## 5. Discussion and Conclusion

Long-term ambulatory fetal monitoring based on noninvasive abdominal measurement of the mECG, fECG, and EHG is an important step closer to becoming feasible when using integrated on-board signal processing to reduce power consumption of wireless transmission to a negligible level. Efficiency of the signal processing tasks is proportional to the number of leads and signal quality, making the choice of optimal electrode number and placement increasingly important. In this paper, the influence of abdominal electrode placement on the measurement quality of mECG, fECG, and EHG signals is explored. The aim is to obtain an electrode grid for ambulatory measurements that guarantees, at all time, optimal electrode pairs for extraction of the fetal heart-rate and uterine contraction frequency in the final stage of pregnancy. Both simulated and measured signals are used to evaluate various electrode placements in an effort to determine the influence of scale and direction on measurement quality for all signals of interest. As the orientation of the fetus is *a priori* unknown and, as a rule, the optimal measurement direction does not equal that of the mECG, an electrode grid is needed for optimal detection of both signals. Based on these results, an electrode grid is proposed, which allows for optimal registration of all signals of interest.

For evaluation of the maternal and fetal ECG, a dipole-based model of the heart using a VCG as input was introduced to simulate the potential due to cardiac activity on the skin surface. The model allows for realistic simulation of the potential at any position on the maternal abdomen and simulation results can be used to determine the influence of electrode placement on the SNR of maternal and fetal ECG signals. Simulation results based on 2 standard 12-lead ECG recordings and 2 in-house abdominal recordings show a strong influence of both the interelectrode distance and orientation of bipolar electrode pairs on the SNR. Depending on the morphology of the ECG, a 90° rotation of the bipolar lead orientation or a reduction of the interelectrode distance by half can lower the SNR by as much as 5 dB.

A set of test measurements was performed to validate and verify the obtained simulation results for the mECG and fECG and obtain quantitative information on the EHG. To this end, a triangular measurement grid was used, which enables measurement of all signals of interest from a plurality of bipolar leads in multiple directions and interelectrode scales. For each of the bipolar derivations, as described in Section 3.2, the SNR was determined for the mECG, fECG, and EHG signals.

For the ECG signals, the influence of interelectrode distance and lead orientation observed in the measurement results clearly show the same trend as the simulation results. The optimal measurement direction on the abdomen for the mECG is horizontal, where an increase in the SNR can be observed with increasing interelectrode distance up to 16 cm. The SNR of the fECG also increases with increasing interelectrode distance, but, contrary to the mECG, it does not show a single electrode pair clearly having the highest SNR for all recordings. This can be related to the changing fetal orientation, resulting in morphological changes in the recorded signal due to a varying angle between the fetal cardiac vector and the bipolar lead. For simulation 1 and patients 2 to 5 in the measurement set, the approximate direction of lead IV can be observed to contain the highest SNR. Simulation 2 and patient 1 show a different optimal direction, as those fetuses displayed a sacrum anterior and occipitoposterior presentation, respectively.

To determine the significance of a change in interelectrode distance or orientation, a *t*-test was performed on every combination of interelectrode distance and orientation with respect to the electrode pair with the highest SNR. A change in interelectrode distance by only 20% produces a statistically significant SNR reduction for the mECG, while for the fECG no significant result was obtained. This fECG result can most probably be ascribed to the low overall SNR, which results in a high relative variation in SNR. A change in measurement orientation by an angle >55° compared to the optimum results in a significant drop in SNR for both ECG signals. Using more than 3 electrodes in a circular layout is therefore not expected to significantly increase the SNR of either the maternal or fetal ECG.

Contrary to the ECG, the SNR of the EHG signal does not consistently improve with increasing interelectrode distance but shows a clear optimum between 8 cm and 14 cm. A *t*-test shows that interelectrode distances of both 4 cm and 16 cm yield a significantly lower SNR. The optimum is introduced because bipolar measurements of propagating signals near the electrodes introduce dips in the signal power spectrum, the frequencies of which are dependent on interelectrode distance and propagation velocity [42]. With an interelectrode distance of 10 cm and an assumed maximal propagation velocity of 10 cm/s [43], the bandwidth of the main lobe of this spatial filter is 1 Hz. Increasing the interelectrode distance beyond 10 cm results in the bandwidth reducing below the EHG frequency band. On average, a horizontal measurement direction of the EHG shows the highest signal quality, although no statistically significant results are obtained. Given the complex underlying mechanisms, different factors, for example, respiration, may contribute to these results [44]. Additionally, the *a priori* unknown origin and direction of propagation of the uterine electrical activity reduce the statistical significance [12]. As the optimal measurement direction during each contraction is different, measuring multiple bipolar lead directions simultaneously is expected to improve reliable contraction detection compared to a single bipolar pair.

**Figure 9:**
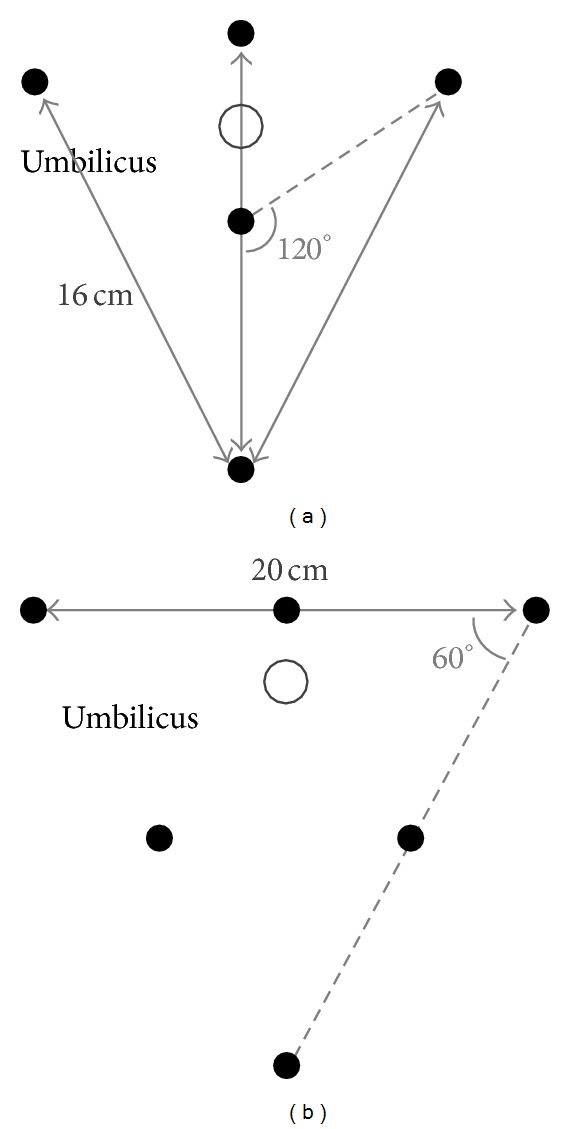
Proposed electrode grids for optimal measurement of the mECG, fECG, and EHG. (a) Grid with five electrodes for measurements close to delivery. (b) Grid with six electrodes for measurements throughout pregnancy.

Based on our results for the mECG, fECG, and EHG, an electrode grid for optimal measurement of all signals of interest can be defined. In the final stage of pregnancy, when the fetus typically has a cephalic presentation, an electrode grid as shown in Figure 9(a) offers optimal measurement results, considering the reduced freedom of movement of the fetus. Earlier throughout the pregnancy the fetus has much more room to move around, and additional fECG measurement directions may be required to guarantee optimal signal quality. Tracking the current fetal position using an algorithm as presented in [45] will allow for optimal electrode selection. An electrode grid with six electrodes at a scale of 20 cm, as shown in Figure 9(b), offers 360° fECG measurements and various EHG signals. While giving optimal results for the EHG measurements, simulation results show that also the maternal and fetal ECG are obtained with a best possible quality given the posed size constraints. Dynamic electrode selection might be used to only record leads with the highest SNR for mECG, fECG, and EHG, reducing power consumption in the analog front-end and digital signal processing.

Future work will focus on methods for efficient and dynamic electrode selection to reduce power consumption in a device aimed at ambulatory pregnancy monitoring. Additional insights could be provided by an extended dataset including measurements at lower gestational ages.

## Figures and Tables

**Table 1:** Mean SNR_*m*_ in dB for both simulations.

		I	II	III	IV	V	VI
Sim. 1	4 cm	**5.8 **	4.8	3.1	3.5	4.6	4.7
	8 cm	9.4	4.1	3.6	7.1	9.7	**10.8 **
	12 cm	**12.4 **	8.1	4.9	6.3	12.1	12.4
	16 cm	**14.6 **	10.9	5.7	7.6	14.3	14.2
	20 cm	**16.1 **	12.0	6.2	8.5	15.5	15.8

Sim. 2	4 cm	5.1	4.3	4.5	4.9	**5.6 **	4.8
	8 cm	8.0	3.8	5.9	7.8	9.3	**9.3 **
	12 cm	**10.9 **	7.8	6.8	9.0	10.8	10.7
	16 cm	**12.6 **	9.8	7.9	9.6	12.1	12.0
	20 cm	**14.3 **	11.2	8.8	10.4	13.6	13.6

**Table 2:** Mean SNR_*f*_ in dB for both simulations.

		I	II	III	IV	V	VI
Simulation 1	4 cm	1.9	2.2	3.2	**3.3 **	3.2	2.5
	8 cm	1.8	2.3	4.2	**4.7 **	4.2	2.0
	12 cm	1.5	2.0	4.0	**5.5 **	3.9	1.9
	16 cm	1.1	1.8	3.5	**6.3 **	3.2	1.2
	20 cm	1.3	1.7	3.4	**6.9 **	3.1	1.4

Simulation 2	4 cm	**2.2 **	2.1	2.0	1.9	2.0	2.1
	8 cm	2.7	**2.9 **	2.1	1.7	1.7	1.9
	12 cm	3.0	**3.3 **	2.6	1.5	1.8	2.3
	16 cm	3.2	**3.9 **	3.0	1.2	1.0	2.0
	20 cm	3.4	**4.4 **	3.3	1.4	1.3	2.6

**Table 3:** Mean SNR_*m*_ in dB for all measurements.

		I	II	III	IV	V	VI
Patient 1	4 cm	−3.9	**3.1 **	1.4	0.5	−3.4	−4.2
	8 cm	**7.2 **	−0.6	3.5	3.0	5.0	−0.2
	16 cm	**11.9 **	7.8	6.3	7.4	10.3	11.2

Patient 2	4 cm	7.3	**8.5 **	7.5	3.9	1.9	5.5
	8 cm	**12.5 **	10.8	11.2	7.1	7.4	6.4
	16 cm	11.5	**12.7 **	12.5	9.9	9.5	11.1

Patient 3	4 cm	**7.9 **	7.4	3.3	1.4	2.3	0.2
	8 cm	**10.5 **	8.9	6.3	4.3	6.0	6.2
	16 cm	**12.3 **	10.8	9.3	8.7	11.4	12.0

Patient 4	4 cm	**6.4 **	4.9	3.3	2.2	4.6	3.5
	8 cm	8.7	6.6	8.5	2.6	8.5	**8.8 **
	16 cm	**14.3 **	13.2	10.8	5.7	12.4	11.6

Patient 5	4 cm	**1.0 **	−0.6	−1.1	−0.3	0.8	0.2
	8 cm	**6.9 **	3.4	3.0	2.9	6.3	6.6
	16 cm	**12.3 **	11.3	10.1	8.5	10.9	11.2

**Table 4:** Mean SNR_*f*_ in dB for all measurements.

		I	II	III	IV	V	VI
Patient 1	4 cm	−2.7	**1.2 **	0.9	0.4	−1.5	−3.0
	8 cm	**1.1 **	−1.1	0.8	0.6	0.9	−0.9
	16 cm	3.7	**5.0 **	4.8	3.9	0.1	0.1

Patient 2	4 cm	−0.1	2.0	**3.1 **	2.2	1.2	−0.8
	8 cm	1.2	3.0	**5.9 **	4.2	4.3	5
	16 cm	−0.7	2.0	5.2	**6.0 **	1.0	−2.5

Patient 3	4 cm	0.9	**2.7 **	1.4	0.7	0.3	−2.2
	8 cm	1.1	4.1	**5.2 **	4.0	1.9	−0.7
	16 cm	−0.7	3.3	5.7	**7.4 **	0.6	−3.4

Patient 4	4 cm	−0.3	−0.7	−0.2	**0.6 **	0.3	−0.6
	8 cm	−1.7	−2.1	−1.1	1.3	**1.6 **	0.6
	16 cm	−6.2	−3.5	2.5	**4.9 **	0.7	−1.7

Patient 5	4 cm	−2.8	−2.1	**1.6 **	1.2	1.4	−0.9
	8 cm	−1.1	−2.3	**6.6 **	5.0	**6.6 **	3.3
	16 cm	−0.6	−4.5	3.0	**8.5 **	8.3	3.4
